# Latent Tuberculosis Infection and Occupational Protection among Health Care Workers in Two Types of Public Hospitals in China

**DOI:** 10.1371/journal.pone.0104673

**Published:** 2014-08-26

**Authors:** Feng Zhou, Li Zhang, Lei Gao, Yibin Hao, Xianli Zhao, Jianmin Liu, Jie Lu, Xiangwei Li, Yu Yang, Junguo Chen, Ying Deng

**Affiliations:** 1 Third Military Medical University, Chongqing, China; 2 Beijing Center for Disease Prevention and Control, Beijing Research Center of Preventive Medicine, Beijing, China; 3 MOH Key Laboratory of Systems Biology of Pathogens, Institute of Pathogen Biology, Chinese Academy of Medical Sciences and Peking Union Medical College, Beijing, China; 4 Zhengzhou Central Hospital, Zhengzhou, China; 5 Henan Provincial Infectious Disease Hospital, Zhengzhou, China; 6 School of Public Health, Zhengzhou University, Zhengzhou, China; Alberta Provincial Laboratory for Public Health/University of Alberta, Canada

## Abstract

**Objective:**

To determine the impact factors of latent tuberculosis infection (LTBI) and the knowledge of TB prevention and treatment policy among health care workers (HCWs) in different types of hospitals and explore the strategies for improving TB prevention and control in medical institutions in China.

**Methods:**

A cross-sectional study was carried out to evaluate the risk of TB infection and personnel occupational protection among HCWs who directly engage in medical duties in one of two public hospitals. Each potential participant completed a structured questionnaire and performed a tuberculin skin test (TST). Factors associated with LTBI were identified by logistic regression analysis.

**Results:**

Seven hundred twelve HCWs completed questionnaires and 74.3% (n = 529) took the TST or had previous positive results. The TST-positive prevalence was 58.0% (n = 127) in the infectious disease hospital and 33.9% (n = 105) in the non-TB hospital. The duration of employment in the healthcare profession (6–10 years vs. ≤5 years [OR = 1.89; 95% CI = 1.10, 3.25] and>10 vs. ≤5[OR = 1.80; 95% CI = 1.20, 2.68]), type of hospital (OR = 2.40; 95% CI = 1.59, 3.62), and ever-employment in a HIV clinic or ward (OR = 1.87; 95% CI = 1.08, 3.26)were significantly associated with LTBI. The main reasons for an unwillingness to accept TST were previous positive TST results (70.2%) and concerns about skin reaction (31.9%).

**Conclusion:**

A high prevalence of TB infections was observed among HCWs working in high-risk settings and with long professional experiences in Henan Province in China. Comprehensive guidelines should be developed for different types of medical institutions to reduce TB transmission and ensure the health of HCWs.

## Introduction

According to the Global Tuberculosis Report from the World Health Organization (WHO), tuberculosis (TB) remains a major global health problem. In 2011, 1.4 million people died of TB, and after the human immunodeficiency virus (HIV),TB ranks as the second leading cause of death from an infectious disease worldwide [1]. The occupational risk of TB among health care workers was demonstrated in the pre-antibiotic era [2], [3]. Indeed, TB infection is a common problem in healthcare facilities [3]. With the emergence of extensively drug-resistant TB, efforts have refocused on TB infection control in healthcare facilities and occupational protection among health care workers (HCWs). The WHO issued a policy on TB infection control in health care facilities in 2009 [4]. Many high-income countries have offered detailed recommendations for latent TB infection (LTBI) screening and preventive therapy among HCWs in their TB guidelines [5]. In these countries, serial surveillance of LTBI and occupational infection control measures have been implemented as regular tasks in the TB infection control programs, and as a result, TB infections have clearly declined in number [6]; however, the situation is very different in low- and middle-income countries because of the high TB burden and limited resources [7], [8], [9], [10]. Most TB control programs in low- and middle-income countries have focused on case detection and treatment using the DOTS strategy. A systematic review of 51 studies showed that the prevalence of LTBI and the annual incidence of TB disease in HCWs ranged from 33% to 79%and 69 to 5,780 per 100,000 in low- and middle-income countries, respectively [11].

China is one of 22 high-TB burden countries with the second largest number of cases [1]. In 2000, a nationwide survey found that 45% of Chinese had TB infections [12]. Reports from several medical institutions have shown that the average prevalence of TB infections has ranged from 49.0% to 60.4% among HCWs using a single-step TST (5 international units; 0.1 ml) [13], [14]. Vaccination against TB with Bacillus Calmette-Guerin (BCG) is routinely offered to Chinese newborns. It has been reported that TST reactions ≥5 mm occur in 69% of the HCWs in Inner Mongolia, China [15]. China has established a TB prevention, treatment, and control system. All types of hospitals or community health service centers have the responsibility of finding, reporting, and referring suspected patients. Local TB centers or designated infectious disease hospitals are responsible for the diagnosis and treatment of TB patients. Local centers for disease prevention and control (CDC) or centers for TB prevention and control are in charge of epidemiologic investigations, data collection, and patient follow-up [16];however, there are no special guidelines for preventing the spread of TB in health care facilities. LTBI surveillance and risk assessment in medical institutions has not been established in China [17]. According to the Fourth National Survey, 91.2% of pulmonary TB patients are first detected in general hospitals [12]. Therefore, general hospitals should pay attention to TB infection control and occupational protection. The aim of our study was to investigate the impact factors of LTBI and the knowledge of TB prevention and treatment policy among HCWs in different types of hospitals and explore the strategies of improving TB prevention and control in medical institutions in China.

## Methods

### Study design and settings

A cross-sectional study was carried out to assess the risk of TB infection and the levels of personnel occupational protection among HCWs who directly engage in medical duties. The following two hospitals were involved in our survey: 1) Zhengzhou Central Hospital is a non-TB, tertiary public and general hospital with 1,400 beds and approximately 1600 HCWs; and 2) Henan Provincial Infectious Disease Hospital is an infectious disease and tertiary public infectious disease hospital with 600 beds and approximately 702 HCWs. Henan Provincial Infectious Disease Hospital is responsible for TB diagnosis, treatment, and management, and is located in Zhengzhou City. TB patients with severe complications who are referred from other cities or prefectures in Henan Province are also admitted to Henan Provincial Infectious Disease Hospital. TB patients are not admitted to Zhengzhou Central Hospital. Patients with suspected TB infections in this hospital will be referred to Henan Provincial Infectious Disease Hospital.

### Participants

The sample size was determined by the number of HCWs who were directly involved in medical duties in the infectious disease hospital because our goal was to assess the influence of medical work and personnel occupational protection of HCWs on TB infection. According to the name list of this hospital, 482 HCWs in the infectious disease hospital were identified to be potential participants for our survey. The categories of departments included outpatient clinics, intensive care units, emergency, internal medicine, infectious disease, Chinese medicine, radiology, stomatology departments, and the laboratory. An equivalent number of medical staff was randomly extracted from each medical department according to the work certification number in a non-TB hospital. All of the HCWs who engaged in medical duties for more than 6 months in these two hospitals were eligible for the personal TB infection risk survey. Each HCW was recruited by their department supervisor and encouraged to complete a self-administrated and standard-structured questionnaire. A TST was performed on all potential participants unless they declined to take or were not available during the study period. The study was approved by the Ethics Committees of the Institute of Pathogen Biology, Chinese Academy of Medical Sciences & Peking Union Medical College. All HCWs provided written informed consent before recruitment.

### Data collection and TST

HCWs were recruited between January and December 2011. A structured questionnaire was used for risk assessment of TB infection among HCWs, including sociodemographic characteristics (e.g., age, gender, ethnicity, education, period of professional work, and employed position), knowledge of TB prevention and control, history of professional work, clinical work, and personal occupational protection. We performed a single-step TST using 5 international units (IU; 0.1 ml) of tuberculin (Chengdu Institute of Biological Products, Chengdu, China), which produces tuberculin from *Mycobacterium bovis* BCG. The TST was administered using the Mantoux method by experienced nurses, and participants returned 48–72 hours after TST placement to obtain results, which were confirmed independently by two clinicians. The diameter of induration size was measured using a standardized ruler and the results were averaged. As the popular BCG vaccination in China, LTBI was determined using a TST induration ≥10 mm as a cut-off point for TST positivity and no TB history was reported by participants in this study. TST-positive participants were required to have an X-ray examination to rule out TB.

### Statistical analysis

Questionnaires were double-entered and compared with EpiData software (EpiData 3.02 for Windows; The EpiData Association, Odense, Denmark). After cleaning, the data were then converted and analyzed using the Statistical Analysis System (SAS 9.2 for Windows; SAS Institute Inc., Cary, NC, USA).

The associations between TB infection and the sociodemographic characteristics, experiences of medical duties, and personal occupational protection, including mask use, washing hands, BCG vaccination, occupational protection training, and physical examination were estimated using univariate logistic regression models. Variables related to LTBI (P<0.05) in the univariate analyses were included in a multiple logistic regression model using stepwise selection of the variables. The knowledge and practices of personal occupational protection in two hospitals was analyzed using Cochran-Mantel-Haenszel test.

## Results

A total of 731 eligible HCWs signed the informed consent and completed the questionnaires (333 from a non-TB hospital and 398 from the infectious disease hospital) between January and December 2011. Five hundred fifty-six of the 731 HCWs completed TSTs or had records of previous positive TST results. Twenty-seven of the 731 HCWs had a history of TB (five from the non-TB hospital and 22 from the infectious disease hospital). Finally, 529 HCWs were used for the risk analyses (310 from the non-TB hospital and 219 from the infectious disease hospital). The age of the participants ranged from 18 to 71 years, with a mean age of 31.4±9.0 years. Of the participants, 97.2% (514/529) were of Han ethnicity, 48.0% (254/529) had bachelor's degrees or higher, 30.4% (160/527) had middle or senior professional qualifications, 90.0% (476/529) lived with family members, and 7.9% (42/529) used cigarettes. Of the participants, 47.3% (167/353) reported having received a BCG vaccination at birth. The TST induration size (mean ± S.D. [mm]) in each hospital was as follows: infectious hospital = 11.9±6.7 mm; and non-TB hospital = 5.2±6.7 mm. The nearest PPD testing was conducted one year ago. The TST results suggested that 43.9% (n = 232) were TST-positive (58.0% in the infectious disease hospital and 33.9% in the non-TB hospital using a TST induration ≥10 mm as a cut-off point; and 75.8% in the infectious disease hospital and 43.9% in the non-TB hospital using a TST induration ≥6 mm as a cut-off point). There was no PPD boosting phenomenon in either hospital. All 232 TST-positive HCWs performed chest X-rays and one TB case was diagnosed. Age, education status, professional qualification, and alcohol consumption were associated with TST-positivity based on univariate analyses (Table 1). One tuberculosis case was diagnosed.

**Table 1:** Associations between demographical characteristics and habit with LTBI§.

Factors	LTBI§	OR (95% CI)*	p value
	n/N 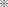 (%)		
**Sex**			
Female	188/442 (42.5)	1.00	
Male	44/87 (50.6)	1.38 (0.87, 2.19)	0.168
**Age**			
<30	112/289 (38.8)	1.00	
30–39	58/125 (46.4)	1.37 (0.90, 2.09)	0.147
≥40	62/111 (55.9)	2.00 (1.28, 3.12)	0.002
**Han Ethnic**			
No	8/15 (53.3)	1.00	
Yes	224/514 (43.6)	0.68 (0.24, 1.89)	0.456
**Education status**			
Under bachelor degree	104/275 (37.8)	1.00	
Bachelor degree and above	128/254 (50.4)	1.67 (1.18, 2.36)	0.004
**Profession qualification**			
Elementary and others	147/367 (40.1)	1.00	
middle/senior	84/160 (52.5)	1.65 (1.14, 2.40)	0.008
**Monthly income (RMB)**			
≤2000	114/271 (42.1)	1.00	
>2000	118/254 (46.5)	1.20 (0.85, 1.69)	0.312
**Residential area**			
Suburban district or rural area	71/161 (44.1)	1.00	
Downtown	161/368 (43.8)	0.99 (0.68, 1.43)	0.941
**Live status**			
Live by oneself or in domitary	20/53 (37.7)	1.00	
Live with family members	212/476 (44.5)	1.33 (0.74, 2.38)	0.345
**Average per-capita living space**			
>20 m^2^	175/382 (45.8)	1.00	
≤20 m^2^	53/130 (40.8)	0.81 (0.54, 1.22)	0.318
**Smoking**			
No	221/487 (43.3)	1.00	
Yes	21/42 (50.0)	1.31 (0.70, 2.46)	0.404
**Drinking**			
No	189/450 (42.0)	1.00	
Yes	43/79 (54.4)	1.65 (1.02, 2.67)	0.041
**Calcium/Vitamin supplement**			
No	188/414 (45.4)	1.00	
Yes	43/114 (37.7)	0.73 (0.48, 1.11)	0.144
**Physical exercise (times per week)**			
≥2	75/184 (40.8)	1.00	
<2	157/345 (45.5)	1.21 (0.85, 1.74)	0.295

§ LTBI, latent tuberculosis infection.

*CI, confidential interval; OR, odds ratio.

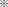
Sum may not always add up to total because of missing data.

Among 529 HCWs, the duration of medical duties ranged from 0.5 to 55.5 years, with a mean duration of 9.7±9.1 years. Of the HCWs, 26.9% (139/516) reported a history of working in TB wards, and 48.2% (255/529) reported intimate contact with TB patients. Univariate analyses suggested that HCWs who worked in TB clinic or wards and HIV clinic or wards, had intimate contact with TB patients, the type of hospital, knowledge of TB treatment and care policy, and the duration of employment as a healthcare professional were associated with TB infection (Table 2).

**Table 2:** Associations between medical works and professional protection strategy with LTBI§.

Factors	LTBI§	OR (95% CI)*	p value
	n/N 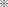 (%)		
**Duration of healthcare profession (years)**			
≤5	88/251 (35.1)	1.0	
6–10	43/81 (53.1)	2.10 (1.26,3.48)	0.004
>10	101/197 (51.3)	1.95 (1.33, 2.85)	0.001
**Type of hospital**			
General hospital	105/310 (33.9)	1.0	
Infectious disease hospital	127/219 (58.0)	2.70(1.89, 3.85)	<0.001
**Department of hospital work**			
Clinical systems	180/418 (43.1)	1	
Ancillary clinical systems	52/111 (46.8)	1.17 (0.77, 1.77)	0.475
**Had ever worked in TB clinic or ward**			
No	147/377 (39.0)	1.00	
Yes	83/139 (59.7)	2.32 (1.56, 3.45)	<0.001
**Had ever worked in HIV clinic or ward**			
No	170/429 (39.6)	1.00	
Yes	60/87 (69.0)	3.39 (2.07, 5.55)	<0.001
**Contact with blood or other body fluid in work**			
No or seldom	113/270 (41.9)	1.00	
Sometime or frequent	119/259 (45.9)	1.18 (0.84, 1.67)	0.343
**Work hours everyday**			
≤8	119/276 (43.1)	1.00	
>8	111/240 (46.3)	1.14 (0.80, 1.61)	0.475
**Consistent mask use in professional work**			
No	68/157 (43.3)	1.00	
Yes	128/281 (45.6)	1.10 (0.74, 1.63)	0.651
**Kind of masks used in medical work**			
mask	135/296 (45.6)	1.00	
Surgical mask	59/131 (45.0)	0.98 (0.65, 1.48)	0.913
N95 respirator	2/7 (28.6)	0.48 (0.09, 2.50)	0.381
**Wash hands**			
No	52/110 (47.3)	1.00	
Wash hands every time	180/418 (43.1)	0.84 (0.55, 1.29)	0.429
**Attending regular physical examination every time**			
No	15/47 (31.9)	1.00	
Yes	217/480 (45.2)	1.76 (0.93, 3.34)	0.083
**Attending infection control training**			
No	17/42 (40.5)	1.00	
Yes	215/487 (44.1)	1.16 (0.61, 2.21)	0.646
**Self-reported using BCG**			
No	14/43 (32.6)	1.00	
Yes	167/353 (47.3)	1.86 (0.95, 3.64)	0.070
Unknown	50/132 (37.9)	1.26 (0.61, 2.62)	0.530
**Intimate contact with TB patients**			
No/Unknown	98/274 (35.8)	1.00	
Yes	134/255 (52.5)	1.99 (1.40, 2.82)	<0.001
**Knowledge of TB prevention and control**			
Unknown	129/309 (41.7)	1.00	
Known	101/218 (46.3)	1.21 (0.85, 1.71)	0.296
**Policy of TB treatment and care**			
Unknown	94/247 (38.1)	1.00	
Known	136/280 (48.6)	1.54 (1.09, 2.18)	0.015

§ LTBI, latent tuberculosis infection.

*CI, confidential interval; OR, odds ratio.

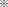
Sum may not always add up to total because of missing data.

The variables associated with TB infection in the univariate analyses (P<0.05) were included in a multivariate logistic regression model. The duration of employment as a healthcare professional (6–10 years vs. ≤5 years [OR = 1.89; 95% CI = 1.10, 3.25]; >10 vs. ≤5[OR = 1.80; 95% CI = 1.20, 2.68]), types of hospital (OR = 2.40; 95% CI = 1.59, 3.62), and ever-employment in a HIV clinic or ward (OR = 1.87; 95% CI = 1.08, 3.26)were associated significantly with TB infection (Table 3).

**Table 3:** The associations of LTBI§ with potential factors in multivariate logistic regression model.

Factors	Multivariate OR (95% CI)*	p value
**Duration of healthcare profession (years)**		
6–10 vs. ≤5	1.89 (1.10, 3.25)	0.021
>10 vs. ≤5	1.80 (1.20, 2.68)	0.004
**Type of hospital**		
Infectious disease hospital vs. general hospital	2.40 (1.59, 3.62)	<0.001
**Had ever worked in HIV clinic or ward**		
Yes vs. No.	1.87 (1.08, 3.26)	0.026

§ LTBI, latent tuberculosis infection.

*CI, confidential interval; OR, odds ratio.

Subset analysis of knowledge about TB and practices of airborne infection control with the LTBI were stratified by employment duration. The depth of knowledge of TB prevention and control and the policy of TB treatment and care, consistent mask use, surgical mask or N95 respirator use, attending physical examination, and infection control training were significantly different between the two types of hospitals (P<0.05) after stratifying the duration of health care professional work. A longer duration of health care professional work tended to decrease the frequency of mask use (Table 4).

**Table 4:** Knowledge about TB and Practice of Airborne Infection Control with the LTBI, stratified by job-duration.

	Non-TB hospital	Infectious Disease Hospital	p value
Factors	n/N 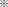 (%)	n/N 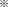 (%)	
**Known the knowledge of TB prevention and control**			
≤5	44/156 (28.2)	39/99 (39.4)	
6–10	17/45 (37.8)	18/37 (46.7)	
>10	56/117 (47.9)	47/84 (56.0)	0.019
**Consistent mask use in professional work**			
≤5	71/116 (61.2)	82/96 (85.4)	
6–10	13/31 (41.9)	27/35 (77.1)	
>10	30/84 (35.7)	58/81 (71.6)	<0.001
**Use surgical mask or N95 respirator in medical work**			
≤5	17/115 (14.8)	52/94 (55.3)	
6–10	8/31 (25.8)	15/35 (42.9)	
>10	10/84 (11.9)	36/80 (45.0)	<0.001
**Wash hands every time**			
≤5	123/156 (78.9)	82/96 (85.4)	
6–10	41/45 (91.1)	27/35 (77.1)	
>10	88/116 (75.9)	67/84 (79.8)	0.794
**Attending regular physical examination every time**			
≤5	119/154 (77.3)	97/99 (98.0)	
6–10	40/45 (88.9)	37/37 (100.0)	
>10	109/117 (93.2)	84/84 (100.0)	<0.001
**Attending infection control training**			
≤5	130/156 (83.3)	96/99 (97.0)	
6–10	39/45 (86.7)	37/37 (100.0)	
>10	111/117 (94.9)	82/84 (97.6)	<0.001
**Known the policy of TB treatment and care**			
≤5	58/156 (37.2)	63/99 (63.6)	
6–10	23/45 (51.1)	28/37 (75.7)	
>10	56/117 (47.9)	57/84 (67.9)	<0.001

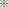
Sum may not always add up to total because of missing data.

Among 712 HCWs who completed questionnaires, 94 provided reasons for declining a TST; 91.5% (86/94) worked in the infectious disease hospital. Previous positive TST results were the most important reason for declining a TST in 70.2% (66/94) of the HCWs. The next most frequent reason for declining a TST was concerns about skin reactions, such as blisters, necrosis, and lymphadenitis in 31.9% (30/94) of the HCWs. Finally, the third most frequent reason for declining a TST was receiving a TST in the past 3 months and anxiety about the psychological burden in 16.0% (15/94) of the HCWs (Table 5).

**Table 5:** Reasons for refusal TST among HCWs (N = 94).

Reasons	Percentage (%)
Previous positive TST results	70.2
Worried about positive reaction	31.9
Did TST in the past 3 months	16.0
Anxious about psychological burden	16.0
Requirement for two visits	9.6
Think oneself can't be infected	4.3

## Discussion

Our study investigated LTBIs using TSTs and the potential impact factors among HCWs who are directly engaged in clinical work in two types of public hospitals in China. Among 529 study participants, TST positivity existed in 58.0% of the HCWs in the infectious disease hospital and 33.9% of the HCWs in the non-TB hospital. Ever-employment in a HIV clinic or ward, duration of employment in the healthcare profession, and types of hospitals were identified as significant predictors for LTBIs. The depth of knowledge of TB prevention and control and the policy of TB treatment and care, consistent mask use, surgical mask or N95 respirator use, attending physical examination, and infection control training were significantly different in the two types of hospitals. A longer duration of health care professional work tended to decrease the frequency of mask use.

Epidemiologic studies of TB infection have been conducted among HCWs using TSTs in many developing countries. A review by Menzie indicated that the median prevalence of LTBI in HCWs was 63%, and varied between33% and79% in low- and middle-income countries [18]. Recent reports from three areas of China also showed a high prevalence of LTBI in HCWs (range33.6%–55.6%) who worked in TB centers or hospitals that admitted TB patients using a TST induration ≥10 mm as a cut-off point or T-SPOT [15], [19], [20]. In our study, the prevalence of LTBIs in a non-TB hospital was similar to a previous report in the general population group ≥15 years of age (45%) when using a TST induration ≥6 mm as a cut-off point [12]. Although it was difficult to classify the source of TB exposure because of the lack of data of LTBI prevalence in the past few years in China, TB infection control still should be emphasized in some departments of non-TB hospitals, such as the clinical laboratory, radiology department, and intensive care unit [21]. In the TB prevention and control system in Henan, provincial infectious disease hospitals are responsible for diagnosis and treatment of various critical infectious disease patients. We noted a slightly higher prevalence of LTBI (58% vs. 51.4%) in the infectious disease hospital compared with a previous study conducted among HCWs working in TB centers in Henan province [19]. This finding maybe induced by the high risk of TB infection when there is frequent contact with complicated TB cases or various infectious diseases with TB co-infections. HCWs play an important role in TB hospital infection control, but few hospitals pay attention to monitoring the status of TB infections among HCWs using TST or other developed diagnostic tests, such as TB-IGRAs (quantitative diagnostic kit for Mycobacterium tuberculosis IFN-γrelease assay) in China. X-rays are commonly used for detecting active TB during regular physical examinations. Further studies should be conducted to collect useful information to support and develop a detailed guideline for occupational prevention and hospital infection control of TB infection in different types of Chinese medical facilities.

More years of medical work have been demonstrated to bean important factor in TB infection among HCWs [18]. We found that the duration of medical work greater than 5 years had a higher risk of TB infection. Mask use was underemphasized in HCWs with a longer duration of medical work in the groups of longer job-duration. TB infection assessment and occupational protection should be of concern in these groups of HCWs.

A previous study reported no N95 respirator was available for HCWs working in TB centers in Henan Province [19]. We found that N95 respirators were still very rarely used in the two types of hospitals in our study. A protective respirator is recommended for use when HCWs directly contact active TB patients in the Guide to the Implementation of Chinese Tuberculosis Control Program [22]; however, many hospitals do not put these suggestions into effect. Therefore, improving the supplemental use of protective respirators should be considered by hospital administration. Subset analysis results also pointed out that the level of knowledge of TB and treatment care policy were low in the two hospitals, especially in the non-TB hospital. The knowledge and policy about TB and personal practice of infection control should be improved in hospitals.

TB is the most common opportunistic infection in HIV/AIDS patients. Our previous review showed that HIV/TB co-infection was 22.8% among AIDS patients in mainland China [23]. Thus, the departments with frequent contact or admission of AIDS patients may have a high risk of hospital infection. TB infection in HCWs who provided services to HIV-infected patients has been reported many years ago [24]. In this study we also found that HCWs who had ever-worked in a HIV/AIDS clinic or ward had a higher prevalence of LTBIs. Interventions that were effective in reducing nosocomial transmission of multidrug-resistant TB in HIV wards have been recommended in 1990 by the CDC in the United States [6]. TB infection control in a HIV ward should also be highlighted in Chinese guidelines. Infection control and health education should be strengthened in some high-risk departments within the hospital.

Serial tests of LTBI are effective occupational protection strategies. China has not recommended routine screening for LTBIs in hospitals and two-step TSTs. The infectious disease hospital in our study has conducted TSTs among HCWs during annual physical examinations for 3years.The non-TB hospital has never conducted TSTs among their HCWs. Considering the acceptance of serial tests and the possible effect of previous TSTs, the TST in our study was combined with the hospital annual physical examination. Even so, some participants still worried about TSTs and declined repeat testing. A description of the reasons for declining TSTs showed skin tests and side effects affected the acceptance of TSTs, especially among HCWs in the infectious disease hospital. Compared with TSTs, IGRAs have been shown to be an institutional cost-saving method and results in higher compliance rates [25], [26]. Guidelines from some countries have recommended IGRAs for serial testing of LTBIs among HCWs [5]. Two commercial systems (T-SPOT.TB, Oxford Immunote Ltd., Oxford, UK and QuantiFERON-TB Gold, Cellestis, Carnegie, Australia) are very expensive in China and therefore unavailable to the general population. Developing guidelines of monitoring LTBIs among Chinese HCWs is urgent. The acceptance of serial testing, the specificity of results, and the cost of tests should be considered.

Several limitations of this study should be kept in mind. First, our study participants do not represent the general HCWs due to the potential limitation of hospital selection and enrollment methods. Second, some bias induced by refusal of TSTs should be considered when interpreting our results. Third, TSTs cannot clearly identify the infection status as current or past infections and are affected by popular BCG vaccinations in China. Potential bias caused by such misclassification cannot be excluded. Fourth, the cross-sectional study design has its limitation on association analysis. Therefore, our results need confirmation by a further large-scale case-control or cohort study.

In conclusion, a high prevalence of TB infection was observed among HCWs working in a high-risk setting and with long professional experience in Henan province in China. The knowledge and policy about TB and personal practice of infection control should be improved in hospitals. Comprehensive guidelines should be developed to ensure the health of HCWs and reduce TB transmission in medical institutions.
